# A Spectroscopic Overview of Intramolecular Hydrogen Bonds of NH…O,S,N Type

**DOI:** 10.3390/molecules26092409

**Published:** 2021-04-21

**Authors:** Poul Erik Hansen

**Affiliations:** Department of Science and Environment, Roskilde University, Universitetsvej 1, DK-4000 Roskilde, Denmark; poulerik@ruc.dk

**Keywords:** intramolecular hydrogen bonds, deuterium isotope effects on chemical shifts, isotope ratios, hydrogen bond energies

## Abstract

Intramolecular NH…O,S,N interactions in non-tautomeric systems are reviewed in a broad range of compounds covering a variety of NH donors and hydrogen bond acceptors. ^1^H chemical shifts of NH donors are good tools to study intramolecular hydrogen bonding. However in some cases they have to be corrected for ring current effects. Deuterium isotope effects on ^13^C and ^15^N chemical shifts and primary isotope effects are usually used to judge the strength of hydrogen bonds. Primary isotope effects are investigated in a new range of magnitudes. Isotope ratios of NH stretching frequencies, νNH/ND, are revisited. Hydrogen bond energies are reviewed and two-bond deuterium isotope effects on ^13^C chemical shifts are investigated as a possible means of estimating hydrogen bond energies.

## 1. Introduction

NH…O,S,N (in the following called NH…X for simplicity) intramolecular hydrogen bonds are very important building blocks in biomolecules, in self-organizing materials, in drugs, in switching molecules and in chemistry as such. Examples are given in this review. As the title indicates, this review is dealing with intramolecular hydrogen bonding. Recent reviews cover this subject [1,2]. Review [1] concentrates on Schiff bases made from salicylaldehyde and TRIS. Rules are set up to predict the predominant tautomer based on linear free energy relationships. Review [2] is focused on aromatic systems such as *o*-hydroxy Schiff bases and Mannich bases and mainly deals with tautomerism. However, in order to make it feasible within the limits of a review of this type, tautomeric systems as such are not dealt with. Nevertheless, NH…X systems are of course one of the two forms of a tautomeric system involving NH and may as such provide useful information in the study of tautomeric systems. Older reviews covering hydrogen bonding and tautomeric systems can also be found [3,4,5]. Energetics are also treated and in that relation the question of hydrogen bond strength will be touched upon. The title may be misleadingly broad. By spectroscopic techniques NMR and infrared spectroscopy are primarily meant, as they are central in these studies. Within NMR, ^1^H-, ^13^C- and ^15^N-chemical shifts, isotope effects on chemical shifts, one-bond NH and long-range coupling constants are included, whereas for IR spectroscopy mainly NH stretching frequencies are explored. Theoretical calculations are included in cases when they supplement experimental results although they are not the focal point for this review. A goal is to give some guidance to which spectroscopic tool to use in a given situation. NH…X hydrogen bonds have been investigated less than OH...X intramolecular hydrogen bonds. For an overview of the latter, see [6].

**Figure 1:**
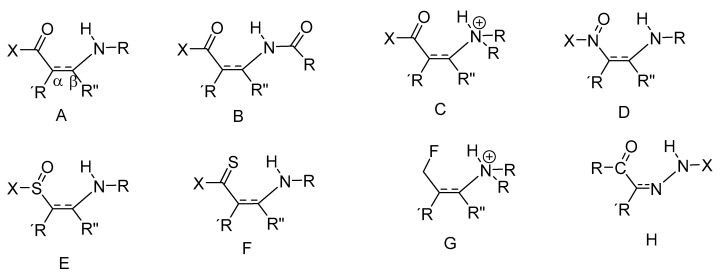
Intramolecular hydrogen bond scheme of RAHB type or charge assisted type. The bond between the α- and the β-carbon can be a double- or an aromatic bond. (**A**) R, R’,R”=H, alkyl or aryl; X=H, C, O, N or S. (**B**) R, R´,R”=H, C,O,N; X=H or alkyl or aryl. (**C**) R=H or C; X=H or C or OR. (**D**) R, R´and R”=H or alkyl or aryl; X=lone pair or O^−^ (nitrogen is positively charged, as it is a nitro group). (**E**) R, R´ and R”=H or alkyl or aryl; X=alkyl or aryl. (**F**) R, R´ and R”=alkyl or aryl; X + R‘=benzene ring. (**G**) R, R´and R”=alkyl. (**H**) R and R´=alkyl, X=Ph.

**Figure 2:**
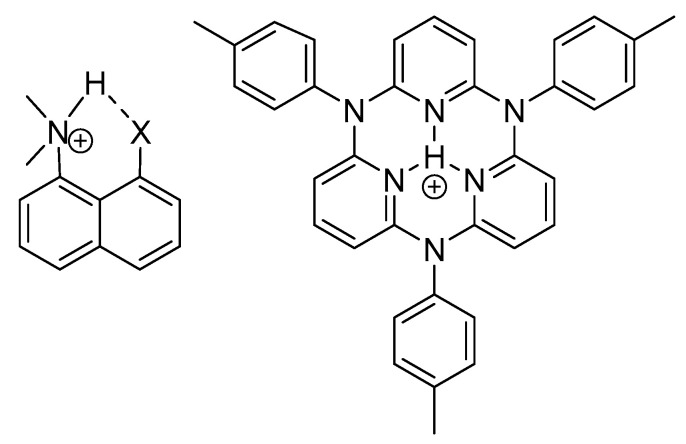
Non-RAHB system. The left hand molecule with X=N(CH_3_)_2_ is the well known DMANH^+^ proton sponge. With X=pyrrole in Figure 4 hydrogen bonding to the π-electron system is found, [7] whereas with X=N(CH_3_)C=OCH_3_ hydrogen bonding to nitrogen have been tested [8]. The right hand molecule, N,N′,N″-tris(p-tolyl)azacalix [3](2,6)pyridine (TAPH), shows an extremely low field NH proton chemical shift of 22.1 ppm [9,10].

Intramolecular hydrogen bonds can be quite different, as seen in Figure 1 and Figure 2. An important feature is the linker between the NH donor and the hydrogen bond acceptor. If this is a double bond or part of an aromatic system, the system has been termed resonance-assisted hydrogen bonding (RAHB) [6] and this clearly influences the type and the strength of the hydrogen bond. In other cases, e.g., proteins, intramolecular hydrogen bonds are not very different from intermolecular ones, except for the fact that the protein may be keeping the donor and the acceptor close to each other. This type of hydrogen bond is clearly very important in proteins, both in defining α-helices, β-sheets and turns. In proteins many hydrogen bonds may be present. Therefore methods to identify specific pairs and to characterize the individual hydrogen bonded pairs is needed. For DNA and RNA the hydrogen bonds are very similar.

In the following, a number of typical hydrogen bond donors and acceptors and pairs of these are compared. It is of course not possible to mention all compounds with intramolecular NH hydrogen bonds. General trends will be given together with typical examples.

## 2. NMR

### 2.1. HN Chemical Shifts

**Figure 3:**
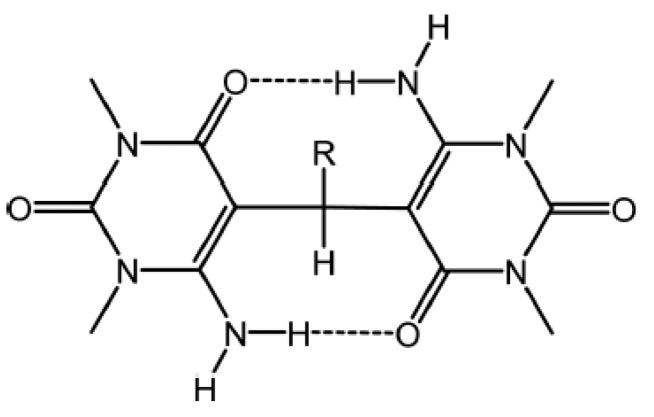
Bis(6-amino-1,3-dimethyluracil-5-yl)-methane derivatives. R being ethyl, pyridine or *p*-dimethylaminopyridine. Taken from [11].

Primary amines may pose a problem in those cases in which rotation around the C-N bond occurs. This leads to averaged NH chemical shifts or as seen later, to averaged one-bond NH coupling constants. In some cases rotation can be stopped at low temperature, as seen e.g., in bis(6-amino-1,3-dimethyluracil-5-yl)-methane derivatives (Figure 3). The chemical shifts of the hydrogen bonded NH protons in these compounds are in the 8–9 ppm range [11].

**Figure 4:**
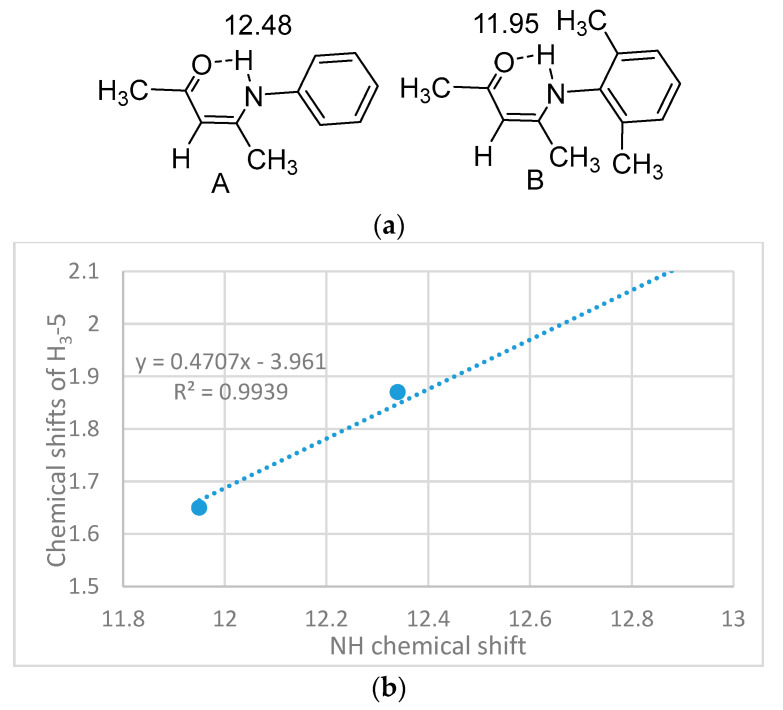
(**a**). Demonstration of possible ring current effects. In A the ring is twisted 34° out to the double bond plane, whereas in B the twist angle is 89°. Data from [12]. (**b**) Plot of ^1^H chemical shifts of CH_3_-5 vs. NH chemical shifts for enaminones with following substituent at nitrogen phenyl substituent, *o*-methyl, *O*,*O*-dimethyl and 4-isopropyl. Data from [13,14,15].

In compounds with aromatic rings close to the NH proton, the NH chemical shift has to be corrected for ring-current effects (for an example of ring current effects see Figure 4) in order to use this to characterize the NH…X hydrogen bond. In A ring a current is present, whereas it is absent in B.

The plot of Figure 4b demonstrates the low frequency shift of both the NH and the CH_3_-5 chemical shift as the phenyl group in the ortho-substituted phenyl ring is twisted.

In β-enaminones (Figure 1A), a hydroxyl group at the phenyl ring next to the carbonyl group clearly competes with the NH group and the NH chemical shift drops to 11.89 ppm [12], whereas an OH group in the ortho-position of a phenyl group at the nitrogen also leads to drop [16], but in this case it is a combination of a twist of the phenyl ring and a field effect caused by the OH group. In case of the *o*-hydroxyphenyl derivative, Rodríguez et al. [17] also report the finding of the keto-form at low concentration in the ^1^H-NMR spectrum but not in the ^13^C-NMR spectrum. *o*-Hydroxyaromatic Schiff bases T are usually either on the OH-form or being tautomeric [18]. However, recently a large number of Schiff bases based on salicylaldehyde and TRIS have been shown to be on the enamine form in the solid state. This is true for the following salicylaldehydes with substituents as follows: 5-nitro, 5-methylcarboxylate, 4-fluor, 4-choro, 4-bromo, 4-methoxy, 4-amino and 5-phenylazo [1]. A few other examples of compounds entirely on the NH form are Schiff bases of 1,3,5-triacyl-2,4,6-trihydroxybenzene [19,20,21], 1,3,5-triformyl-2,4-6-trihydroxybenzene [19], of gossypol [22,23] or more recently of primarily on the NH form (2-(anilinemethylidenen)cyclohexane-1,3-dione) [24].

**Figure 5:**
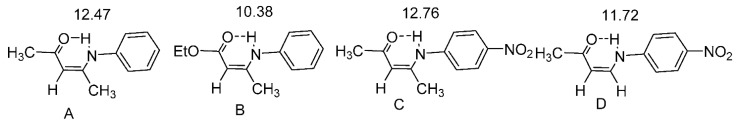
Comparison of different acceptors (**A**,**B**) and demonstration of steric compression (**C**,**D**). The numbers are NH chemical shifts in ppm. A and B from [15,26]. For the corresponding N-methyl derivatives, the chemical shifts are 10.90 ppm and 8.55 ppm. (**C**) is from [12] and (**D**) from [27].

A classic comparison is that of hydrogen bonding involving a ketone or an ester is seen in Figure 5A,B. Another comparison can be found in Ref. [25].

**Figure 6:**
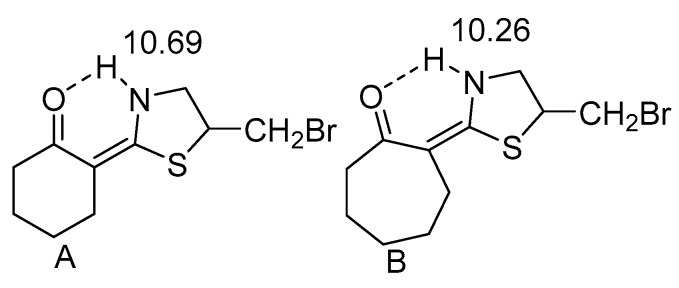
Illustration of the importance of ring size. Taken from [28]. C shows the effect of a six-membered ring. Taken from [29]. The numbers are the NH chemical shifts in ppm.

It is obvious that substitution at nitrogen plays a role. Furthermore, steric compression is also an important feature as seen by comparing the 3-methyl derivative C with the corresponding non-substituted compound D. The introduction of the nitro groups leads to a slightly more acid NH group and to a stronger hydrogen bond (A vs. C). By comparison of the following compounds Figure 6, it is also clear that ring-size plays a role.

**Figure 7:**
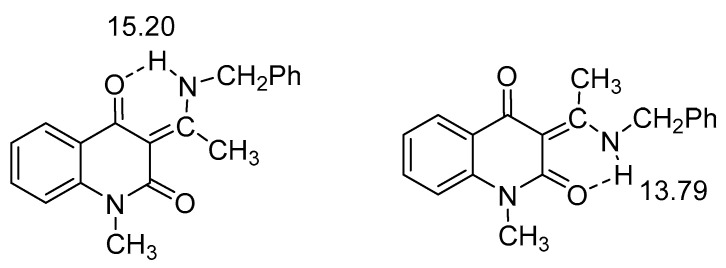
Comparison of ketone and amide groups as acceptors. Data from [30]. The numbers are the NH chemical shifts in ppm.

Another comparison is made in Figure 7, in which the NH is hydrogen bonded to a carbonyl group or to an amide group. The acceptor amide as acceptor clearly leads to the weaker hydrogen bond.

**Figure 8:**
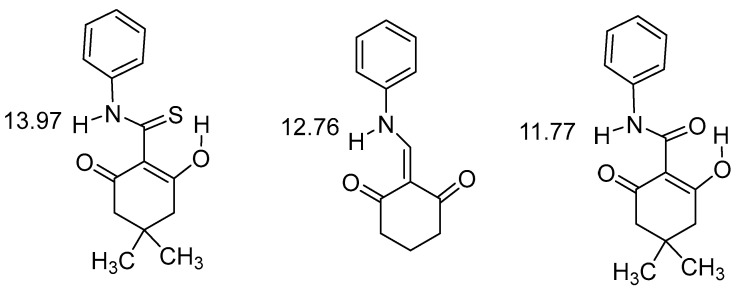
Comparison of amides and thioamides as donors. The numbers are the NH chemical shifts in ppm. For the amide and thioamide the methyl derivatives have chemical shifts of 9.69 and 12.20 ppm. Taken from [31]. The amine is from [24].

Amides and thioamides as donors are compared in Figure 8 [31].

**Figure 9:**
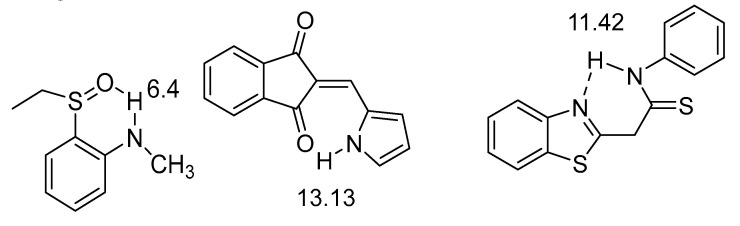
Hydrogen bonding with different motifs. The numbers are NH chemical shifts in ppm. The sulfoxide is from [32]. The indole derivative is from [33]. Other similar motifs is seen in this reference. The benzo[d]thiazol is from [28].

It is seen from Figure 6 that the thioamide is the strongest donor followed by the amine and in third place the amide. Hydrogen bonding to a S=O acceptor is demonstrated in Figure 9.

**Figure 10:**
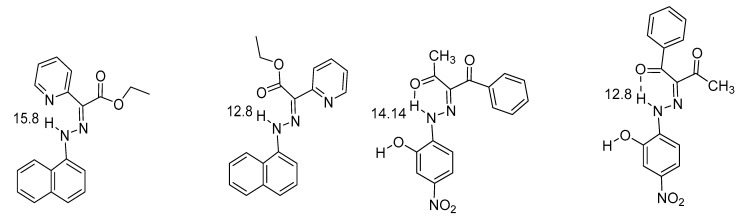
Comparison of rotamers. The numbers are NH chemical shifts in ppm. The chemical shifts are from [36]. The NH chemical shifts for the corresponding benzene derivative (benzene instead of naphthalene) are 14.6 ppm instead of 15.8 ppm. [34] If the ring is a 8-benzoquinoline the chemical shift is 15.36 ppm [37].

The low chemical shift of the benzo[d]thiazol is due to the lack of conjugation between the donor and the acceptor. In Figure 10 are comparisons again done between different acceptors. NH chemical shifts may for hydrogen bonded hydrazo compounds reach values as high as 15.8 ppm when the NH is hydrogen-bonded to a pyridine nitrogen. In the minor isomer, in which the NH is hydrogen bonded to an ethyl ester group, the chemical shift is 12.8 ppm [34]. In the other pair it is obvious that the stronger hydrogen bond is to a CH_3_C=O rather than to a PhC=O. The ability of aromatic nitrogens to form hydrogen bonds is also demonstrated in (*Z*)-5-((phenylamino)methylene)quinoxaline-6-(5*H*)-one, 13.15 ppm in DMSO-d_6._ This value drops to 11.15 ppm in in (*Z*)-4-((phenylamino)methylene)thiadiazol-5-(4*H*)-one, which has a sulphur instead of a CH_2_=CH_2_ unit and hydrogen bonding nitrogen is now part of a five-membered ring [35]. This isomer is with hydrogen bonding to nitrogen is the minor form. The authors discuss hydrogen bonding in terms of quasi-aromaticity.

**Figure 11:**
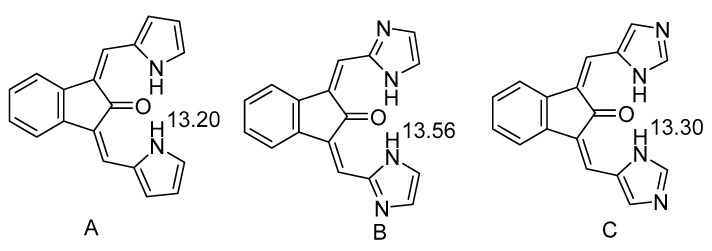
Bifurcated hydrogen bonds. Taken from [40]. The numbers are the NH chemical shifts in ppm.

Pyrroles can also be hydrogen bond donors as seen in a series of compounds (Figure 11). The NH chemical shifts vary from 10.16 to 13.07 ppm [38]. In a similar case but with an OH group as the acceptor, and two pyrroles present, one hydrogen bonded the other not, the chemical shift drops to 9.39 ppm [39].

Bifurcated intramolecular hydrogen bonds were found in azoylmethylidene derivatives of 2-indanone (Figure 11) [40]. Similar kind of molecules have been used to establish a correlation between NH chemical shifts and hydrogen bond energy (see Section 3). It can be seen how the nature of the donor influences the chemical shifts slightly. In case of C the corresponding compound with only one intramolecular hydrogen bond has a chemical shift of 13.73 ppm illustrating the effect of bifurcation. The benzene ring seems to have little effects as the compound corresponding to A simply with the cyclopentanone unit also has a chemical shift of 13.20 ppm. However, by inserting a cyclohexanone ring the chemical shift drops slightly [38]. A different kind of bifurcation can be found in 5-(4-substituted phenylazo)-1-carboxymethyl-3-cyano-6-hydroxy-4-methyl-2-pyridones [41].

**Figure 12:**
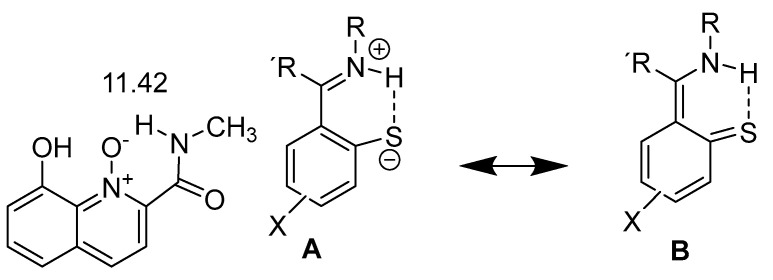
The amino N-oxide demonstrates hydrogen bonding to a charged acceptor. Taken from [42] For the thio-Schiff base resonance forms are demonstrated. Taken from [43]. The number is the NH chemical shift in ppm.

Not so common motifs are seen in Figure 12. The question is if the rather high chemical shifts in the N-oxide are caused by a strong hydrogen bond or an electric field effect from the N^+^-O^−^ bond. A fact is that the deuterium isotope effects on C=O and CH_3_ carbon chemical are rather small [42] (for a general discussion of isotope effects see Section 2.4.1). The thio-Schiff base in Figure 12 is drawn as a neutral molecule and as a zwitterionic structure. The latter contributes quite considerably. The NH chemical shifts vary from 18.06 ppm for R and R′ = CH_3_ to 19.26 ppm for R= PhN(CH_3_)_2_ and R′=CH_3_ [43] or 17.33 ppm for CH_3_=PhCH_3_, R′=CH_3_ or 18.2 ppm for R=PhOCH_3_ and R′=CH_3_ [44]. Similar values were also obtained for derivatives in which R′ is H [45].

**Figure 13:**
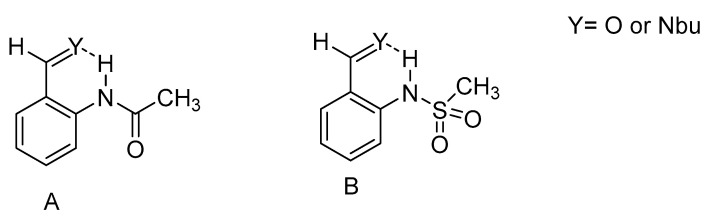
Comparison of different amides as acceptors. In A Y=O the NH chemical shift is 10.97 ppm, whereas when Y=N-butyl it is 18.83 ppm. In B the NH chemical shift is 10.50 vs. 12.74 ppm. Taken from [46].

In Figure 13 is seen a comparison of an aldehyde or an imine as acceptor and a donor being either an amide or a sulfamide. The imine is the best acceptor and as the amide is a better donor than the sulfamide.

**Figure 14:**
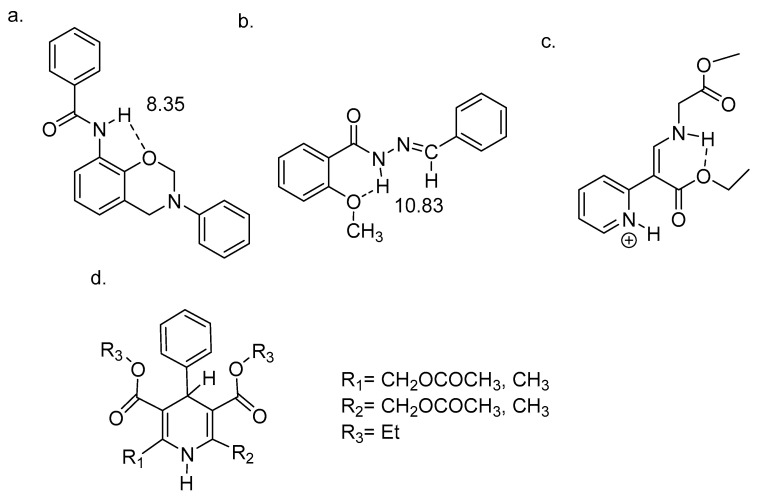
Hydrogen bonding to single bonded oxygen. (**a**). Benzoxazine with intramolecular hydrogen bonding from [47]. (**b**). N’-benzylidenbenzohydrazide from [48]. (**c**). Protonated enamine molecular switch from [49]. (**d**). 1,4-dihydropyridine derivatives from [50]. The numbers are the NH chemical shifts in ppm.

Hydrogen bonding is clearly weaker when the hydrogen bonding is to a sp^3^ hybridized oxygen and the hydrogen system is a five membered ring as demonstrated in the benzoxazine in Figure 14. Although the hydrogen bond is not so strong it is concentration independent [47].

In the case of N′-benzylidenbenzohydrazide, as seen in Figure 14b, a weak hydrogen bond to the methoxy group is formed. This is clearly stronger than when a classic amide is the hydrogen bond donor. In Figure 14c an amine is hydrogen bonded to an ester oxygen [49].

Charged species show large NH chemical shifts. The protonated DMAN’s are tautomeric, but as long as they are symmetric this will not influence the NH chemical shifts. For a review of these see [51]. Very recently an amide type has been investigated. The R substituent has a small effect [8]. Recently motifs A and C have been combined [52].

**Figure 15:**
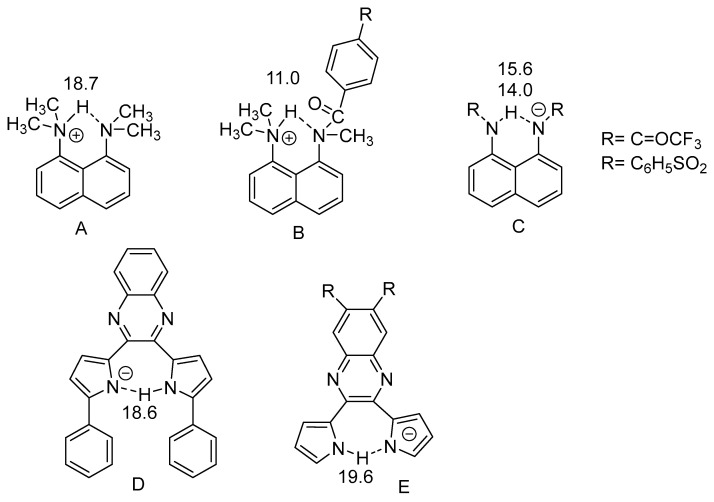
Hydrogen bonding of DMAN types (**A**–**C**). Counter ions are left out, but the numbers vary slightly with the counter ion. (**C**,**D**) are hydrogen bonding to a negative acceptor. Numbers are NH chemical shifts. (**A**) from [53], (**B**,**C**) from [54]. (**D**) from [55]. (**E**) from [56].

It is obvious from the chemical shifts seen in Figure 15 and in Figure 2, that the charged systems have high NH chemical shifts. These systems are typically tautomeric and show strong intramolecular hydrogen bonds.

Motifs involving urea can be found in a review by Osmialowski [57]. Urea is versatile, as it can act both as an amide type donor and acceptor. The intramolecular nature of the hydrogen bonds were among other things established by measuring the temperature dependence. Temperature dependence was also used to distinguish between NH and OH hydrogen bonds [16]. However, this technique is by no means a reliable tool [58]. Oxamides and thioamides NH temperature coefficients have also been investigated to distinguish intra from inter molecular hydrogen bonding [59].

A number of non-RAHB cases are seen in Figure 14. In addition, proteins often offer many intramolecular hydrogen bonds (for use of coupling constants see Section 2.2). To use NH chemical shifts these should be corrected. This subject has been treated in dipeptides by Scheiner [60].

The results of Figure 4, Figure 5, Figure 6, Figure 7, Figure 8, Figure 9, Figure 10, Figure 11, Figure 12, Figure 13 and Figure 14 can be summarized in the following way: thioamides seem to be better than hydrazo groups as donors. They are slightly better than aromatic amines, which again are better than aliphatic amines, amides and sulfamides in that order. The pyrroles are not so easy to fit into this scheme. Even when the hydrogen bond is part of a seven membered ring, they are clearly forming strong hydrogen bonds. As acceptors thiones are better than pyridines and other nitrogen containing rings, imines are better than ketones, which are better than amides and esters. Sulfoxides are rather poor. Single bonded oxygens are even poorer. For charged systems the number of cases is limited, but seems to follow the neutral ones. However, the chemical shifts are much higher both in cases with the donor being positive charged or the acceptor being negatively charged as compared to the neutral cases.

### 2.2. Coupling Constants

Two types of couplings are immediately useful, ^1^*J*(N,H) and for derivatives of aldehydes, ^3^*J*(NH,CH). ^1^*J*(N,H), one-bond hydrogen nitrogen couplings show often a numerical value of around 90 Hz. This coupling is of course negative. Dudek and Dudek showed a small difference between hydrogen bonded and non-hydrogen bonded cases [61]. ^1^*J*(N,H) couplings have also been calculated by DFT methods. A recent study optimized for secundary amines the functional and basis set as follows: B3LYP/6-311++G** for structure optimization in chloroform (PCM approach) and APFD/6-311++G**(mixed) for calculation of ^1^*J*(^15^N,H) coupling constants. A very good agreement with experimental values was found. The shorter the bond the larger the coupling constant [62]. A number of useful trends were found to complement the not so many experimental data. Using a simpler basis set, B3LYP-6-31G, it was found that one has to distinguish between primary and secondary amines. For the primary amine cases dissolved in a hydrogen bonding solvent like dimethylsulfoxide, a sulfoxide molecule has to be hydrogen bonded to the “free” NH in order to obtain good results [3].

The ^3^*J*(NH,CH) coupling is for a non-tautomeric case close to 12 Hz [61]. The observation of a coupling of this magnitude or ^1^*J*(N,H) of around 90 Hz is a clear indication that one is actually dealing with a NH…X hydrogen bond and not with an OH…X one or a tautomeric system. The access to reliable calculations of ^1^*J*(N,H) enables one to calculate values for tautomeric systems, but also to estimate the influence of substituents.

### 2.3. Non-RAHB Cases. Couplings across Hydrogen Bonds

The NH…X bond is central both to proteins, DNA and RNA. A breakthrough was the observation of couplings across hydrogen bonds in RNA [63]. The presence of large *J* couplings (6–7 Hz) between the hydrogen bond (H-bond) donating and accepting ^15^N nuclei in Watson–Crick base pairs in double-stranded RNA was found [64]. For proteins, both in α-helices and in β-sheets they are ubiquitous. These hydrogen bonds are generally not very strong. However, in the stronger cases very interesting coupling constants across hydrogen bonds between ^15^N and ^13^C=O (also referred to as C′) have been observed [65,66] enabling pairing of hydrogen bond donors and acceptors. Correlations with bond lengths ^3h^*J*_NC′_ = −59000 exp(−4*R*_NO_) ± 0.09 Hz, or *R*_NO_ = 2.75 − 0.25 ln(−^3h^*J*_NC′_) ± 0.06 Å have been established [67]. Normally such coupling can only be observed in proteins below 10 kD. However, with perdeutration ^3h^*J*_NC′_ scalar couplings across hydrogen bonds could be observed in the uniformly ^2^H/^13^C/^15^N-enriched 30 kDa ribosome inactivating protein MAP30 [68].

**Figure 16:**
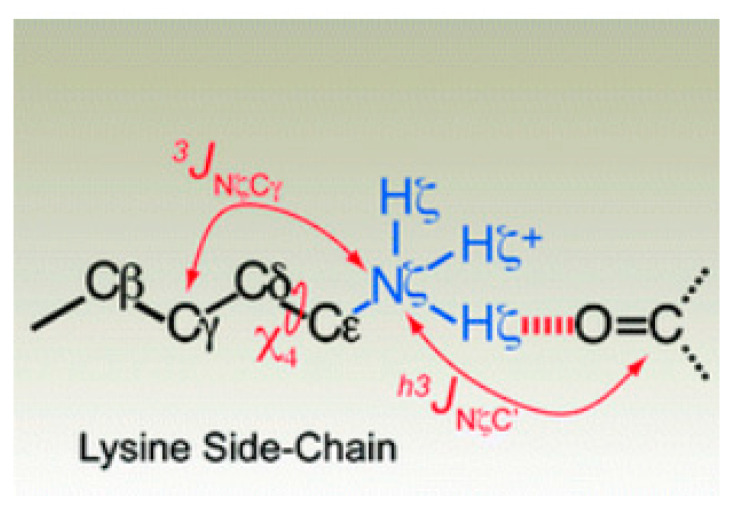
Coupling from at carbonyl carbon to a side-chain lysine N via the hydrogen bond. Taken from [69] with permission from The American Chemical Society.

A study of lysine interactions in ubiquitine with carbonyl backbone revealed that the NH_3_^+^ groups of Lys29 and Lys33 exhibit measurable ^h3^*J*_NζC′_ couplings arising from hydrogen bonds with backbone carbonyl groups of Glu16 and Thr14, respectively. For an example see Figure 16. ^3^*J*_NζCγ_-coupling constants could also be measured, these together with relaxation studies showed that the NH_3_^+^ groups are involved in a transient and highly dynamic interaction [69].

**Figure 17:**
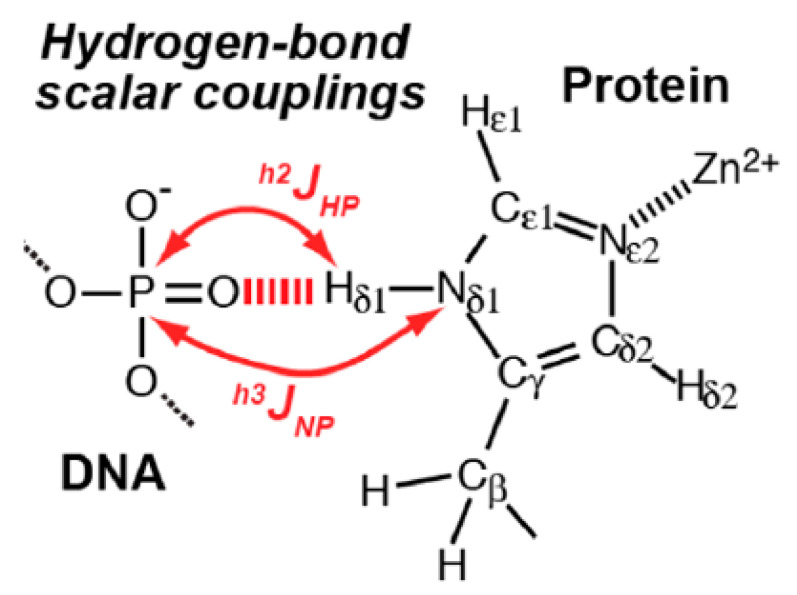
Hydrogen bond scalar coupling involving a phosphate and a histidine. Taken from [71] with permission from the American Chemical Society.

A plot of ^1^*J*(N,H) vs. dN*H* for DNA and RNA demonstrated that the N1…N3 hydrogen bonds are stronger in dsRNA A:U than in dsDNA A:T bases pairs [70]. Both two-bond ^1^H-^31^P and three bond ^15^N-^31^P couplings have also been seen from a histidine to the phosphate group of DNA in a zink finger (see Figure 17) [71].

A very large N…N coupling of 40 Hz through a hydrogen bond is seen in the compound in Figure 15E. The N..N distance is calculated as 2.54 Å. The coupling is the largest of this kind so far reported in a symmetric system [56].

Couplings across hydrogen bonds have also been calculated and summarized by Del Bene [72]. The couplings are dominated by the Fermi contribution and depends on the distance between the heavy atoms. Relationships are noted between hydrogen bond type, X–Y distances, NMR spin–spin coupling constants, and infrared proton-stretching frequencies. This also nicely reflects the experimental findings.

### 2.4. Isotope Effects on Chemical Shifts

Three different types of deuterium isotope effects on chemical shift are useful, ^n^ΔC(ND), ^1^ΔN(D), ^n^ΔH(ND) and in principle ^n^Δ^17^O(ND) in the study of intramolecular hydrogen bonds [73]. Isotope effects is in the present review defined as: ^n^Δ = δX(H) − δX(D).

#### 2.4.1. RAHB Cases (a “Double Bond” Connecting Cα and Cβ)

**Figure 18:**
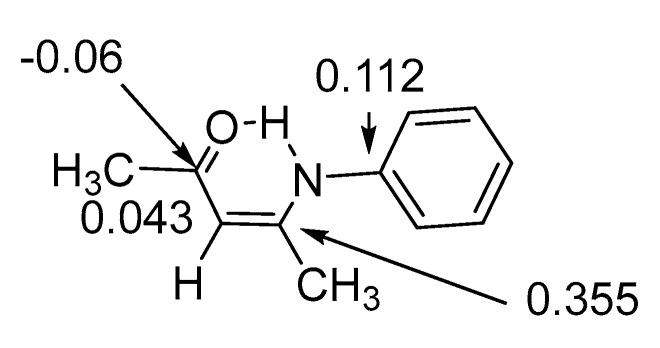
Deuterium isotope effects on ^13^C chemical shifts in ppm. Taken from Ref. [12].

^n^ΔC(ND) were early on studied in enaminones [74]. Later this study was extended 12,18,32,75,76]. An advantage of studying enaminones is that a number of these may exist both in an *E*- and a *Z*-form. The former without an intramolecular hydrogen bond, the latter with and thus giving a genuine reference compound. This kind of study clearly showed that the two-bond deuterium isotope effect on ^13^C chemical shifts, ^2^ΔC(ND), are larger in the intramolecular hydrogen bonded case (Figure 18). This was ascribed to resonance assistance. Having e.g., a substituent at the C-β carbon can introduce steric strain. This will lead to a larger two-bond deuterium isotope effect as seen by comparing number from Figure 14 and Figure 16 (see later) and to a stronger hydrogen bond. An interesting feature in such systems is also the observation of isotope effects at the carbon involved in the intramolecular hydrogen bond and even the carbon attached to the carbonyl group and beyond (see Figure 14) making this a tool for establishing pairs of hydrogen bonds in systems with several possibilities.

**Figure 19:**
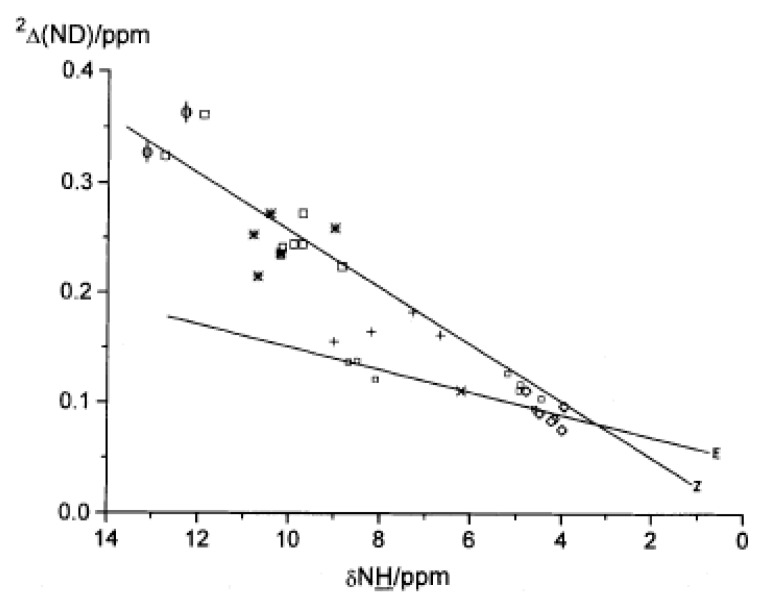
Plot of two-bond deuterium isotope effects on ^13^C chemical shifts vs. NH chemical shifts for enamines. Open squares (*Z*)-enaminones, closed squares (*E*)-enaminones, + enamino esters, * (*Z*)-nitroenamines, crosses (*Z*)-sulphinylenamines and diamonds (*E*)-sulphinylenamines, φ indicates N-phenyl groups. Taken from [32] with permission from Wiley.

Isotope effects have often been plotted vs. XH chemical shifts. [75] In the present case, two-bond deuterium isotope effects (TBDIE) are plotted vs. NH chemical shifts (Figure 19). It is clear that *E*- and *Z*-derivatives can be distinguished. The correlation covers enaminones, nitro- and sulphinyl derivatives [32].

**Figure 20:**
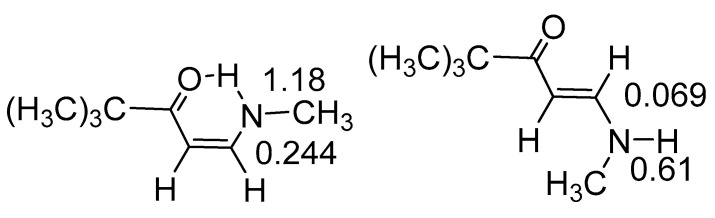
One-bond deuterium isotope effects on ^15^N chemical shifts and two-bond deuterium isotope effects at carbon in ppm. From [76].

One-bond deuterium isotope effects, ^1^ΔN(D), depend strongly on hydrogen bonding (the geometry) and related to that RAHB (see Figure 20). [32]

#### 2.4.2. Non-RAHB Cases

Deuterium isotope effects on ^15^N chemical shifts have been studied in the mono anion of 2,3-dipyrrol-2-ylquinoxaline and its 6,7-dintro derivative (see Figure 15E) [56]. The effects 1.13 ppm and 0.88 ppm (signs have been changed from the original publication) are rather large and indicate a strong hydrogen bond.

Deuterium isotope effects on ^15^N and ^1^H chemical shifts have been used to judge whether certain salt bridges, which are observed in the solid also exist in solution. An example is protein G, B1 domain. The isotope effect demonstrated that two salt bridges found in the X-ray structure did not exist in solution [77]. This approach was also used in Barnase [78]. Both types of isotope effects have also been treated theoretically [79].

**Figure 21:**
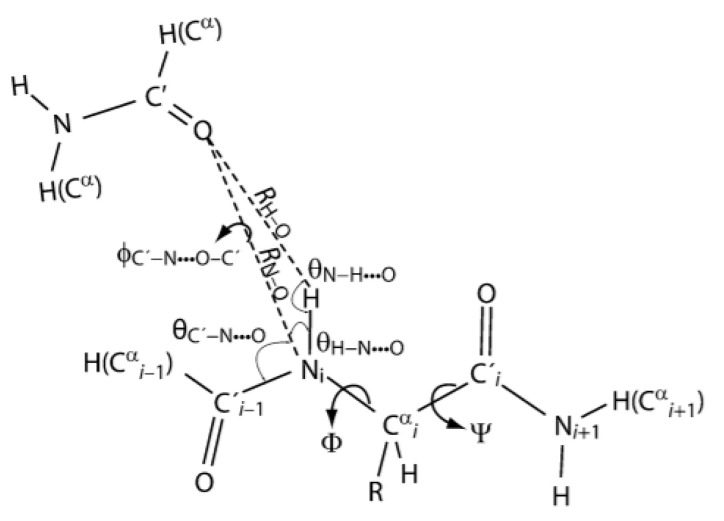
Angles and distances for an inter-chain hydrogen bond. Taken from [80] with permission from Springer.

In ubiquitin the one-bond deuterium isotope effects of hydrogen bonded NH of the back-bone is correlated to the back bone angles and the angle between the acceptor oxygen and the NH bond (Figure 21) [80].

**Figure 22:**
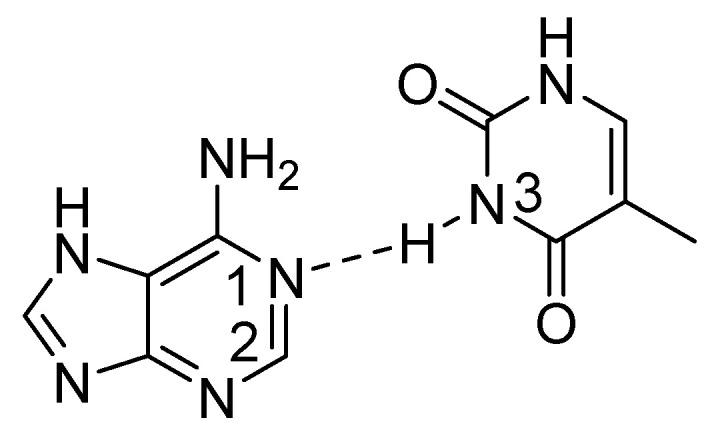
Example of hydrogen bonding in an adenine:thymine base pair.

In DNA and RNA through hydrogen bond isotope effects on chemical shifts can be seen from H-3 to C-2 between adenine and thymine respectively uracil. The isotope effects on chemical shifts are found to be sensitive to the N1-N3 distance suggesting that the isotope effect is sensitive to hydrogen bond strength (see Figure 22) [81,82].

**Figure 23:**
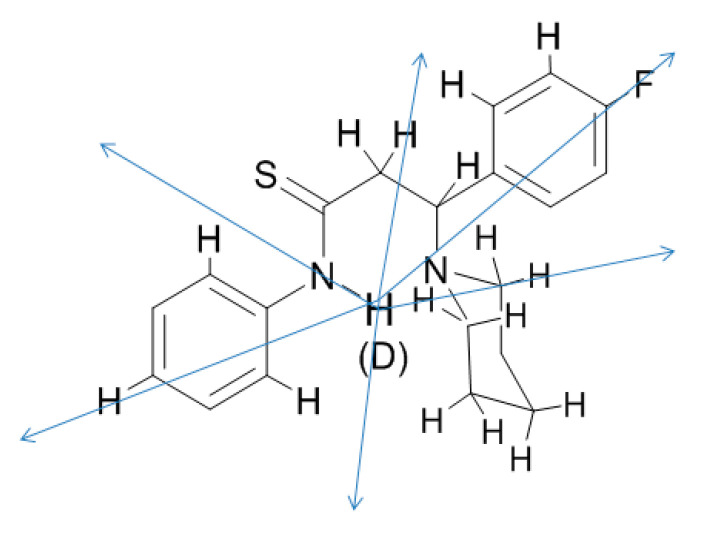
Example of electric field isotope effect through space in N-substituted 3-(cycloamino)thioproionamides.

Very long range isotope effects due to deuteriation at NH have been observed in N-substituted 3-(cycloamino)thioproionamides [83]. These effects were ascribed to electric field effects (Figure 23). The use of nuclei with a large chemical shift range like ^19^F makes this kind of effect very useful even for weaker hydrogen bonds.

#### 2.4.3. Primary Isotope Effects

^P^Δ^1^H(ND), and ^P^Δ^1^H(NT), in which the NH is replaced by either deuterium or tritium may also be used to gauge intramolecular hydrogen bonding. Only a few examples are available [84].

**Figure 24:**
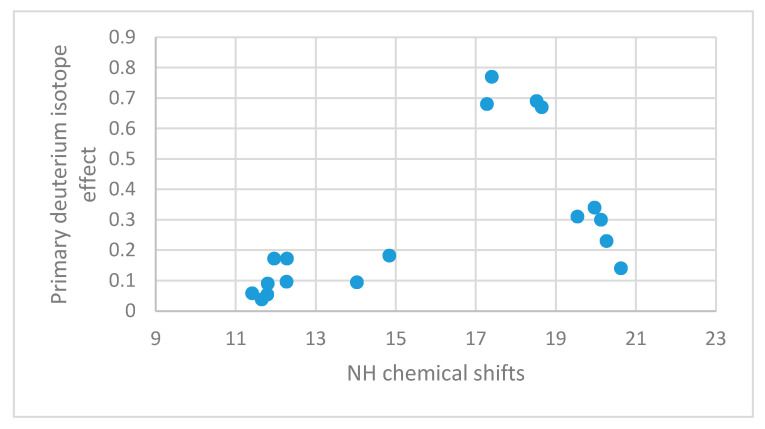
Plot of primary deuterium isotope effects vs. NH chemical shifts. Data from [56,85].

Protonated DMAN’s (DMANH^+^) (Figure 2) show tautomerism. However, for symmetric compounds this will not influence the primary isotope effects [56]. For DMANH^+^ itself the ^P^Δ^1^H(ND), is 0.69 ppm. Similar values were found for a 4,5-CH_2_OCH_2_ derivative as well as the one in which the methyl groups are changed to ethyl groups. However, for 2,7-substituted derivatives the values are much smaller, dichloro, dibromo, bisdimethylamino, dimethoxy and ditrimethylsilyl gave values of 0.30, 0.23, 0.34, 0.31 and 0.14 ppm. [85] The much smaller values in these derivatives were ascribed to a buttressing effect leading to a shorter N…N distance [86]. These data and more together with data from Ref. [84] are plotted in Figure 24. Interesting points falling in-between at chemical shifts 14.03 and 14.84 ppm are N-phenyl derivatives, so these chemical shifts must be corrected for ring current effects. Others are also N-phenyl derivatives, but substituted in the 2-position so the rings are twisted heavily out of the double bond plane and thereby reducing the ring-current effects. The primary isotope effects were also related to IR isotope ratios [73].

As the review deals with NH hydrogen bonds the obvious isotope to use is deuterium. Secondary deuterium isotope effects over two-bonds tell in a qualitative way about the hydrogen bond strength. The larger the isotope effect, the stronger the hydrogen bond. For a relation to hydrogen bond energy see Section 3. In case of one bond deuterium isotope effects on ^15^N two different scenarios are found. In RAHB cases the isotope effect increases with increasing hydrogen bond strength, whereas for intramolecular hydrogen bond cases with no direct connection between donor and acceptor (like in DNA) the magnitude of the isotope effect decreases as the distance between heavy atoms decreases.

The primary isotope effects deuterium isotope effects show a more irregular behavior. It would be interesting in the future to compare primary isotope effects for the systems shown above with the one bond secondary isotope effects on ^15^N.

## 3. Energy

Hydrogen bond energies for intramolecular hydrogen bonds of the RAHB type are difficult to determine experimentally. Nevertheless, experimental values are necessary in order to have a gauge for theoretical calculations. Spectroscopic data can be useful in this context. The question of calculating the hydrogen bond energy for NH…X bonds were treated by Reuben [87]. He suggested to calculate the hydrogen bond energy by extending the equation originally suggested by Schaefer [88] (in this case based on OH…O intramolecular hydrogen bond). It is important to remember that Schaefer said a tentative equation and “rather involved but approximate calculations of electric field effects of the chemical shifts of the hydroxyl proton in intermolecularly hydrogen bonded phenol predict a very nearly linear relationship between the chemical shift and the energy”.

The energy was obtained from a correlation with NH chemical shifts:

ΔδNH = −1.06 + E_H_ (1)

ΔδNH is in the Reuben case referred to the NH chemical shift of N-methylaniline in CDCl_3._ Chiara et al. [89] used these equations to obtain hydrogen bond energies of nitro-substituted enaminones of the order of 29 to 34 KJ/mole. A very comprehensive overview is given by Afonin et al. [33] but mostly for weak interactions. Afonin et al. used a slightly different correlation:

E_HB_(Δδ) = Δδ + (0.4 ± 0.2) energy in Kcal/mol

Afonin et al. [33] used in their study the NH donor in a pyrrole ring together with OH…X and CH…X intramolecular hydrogen bond. In this case the reference compound was pyrrole with a chemical shift of 9.25 ppm. Recently other theoretical approaches have been used. Tupikina et al. used ^1^H chemical shifts of NH_2_ groups using the non-hydrogen bonded NH as reference for aniline derivatives and looking mainly at intermolecular hydrogen bonds. Unfortunately, the only RAHB system, an *o*-amino Schiff base, falls off the correlation line obtained [90].

**Figure 25:**
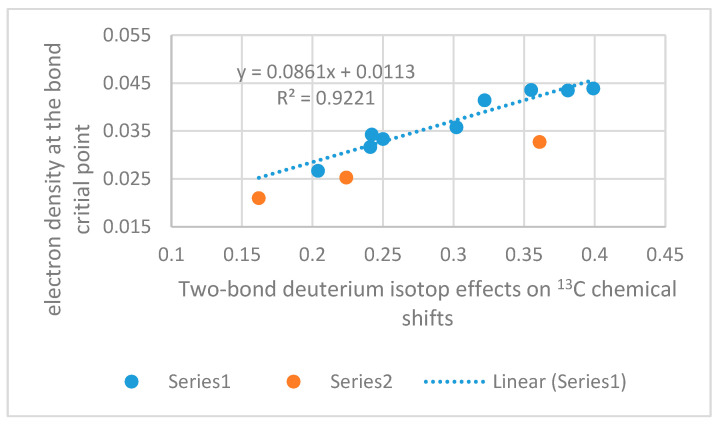
Plot of electron densities at the bond critical point vs. two-bond deuterium isotope effects on ^13^C chemical shifts in ppm. Series 1 include linear compounds with ketones and nitro groups as acceptors, Series 2 include cyclic compounds both 5- and 6-membered rings, ketones and esters. Isotope effects in ppm from [76,89,102,103]. The ring critical points were calculated using the B3LYP/6-311++G(d,p) functional and the AIM program.

The hydrogen bond and out scheme [91] used for intermolecular hydrogen bonds cannot really be used for NH…X intramolecular hydrogen bonds. A scheme has been set up by Jablonski et al. [92] for NH_2_ groups as donors and later used for APO and 3-methyl APO [93]. A simpler method is to use the “in” and 90 degrees approach in which the energy of the latter is subtracted from the hydrogen bonded one. An example is hydrazone switches. A correlation is found between the hydrogen bond energy and the long range NH coupling across the hydrogen bridge [94]. A different method is to use the electron density at the bond critical point as suggested by Rozas et al. [95]. Using this method for 3-aminopropenal Vakili et al. [93] found 26.6 KJ/mol in good agreement with those of Jablonski et al. using MP2/6-31G** and MP2/6-311++G** [92]. The electron density at the bond critical point is used to estimate the hydrogen bond strength in a number of strategic intramolecular hydrogen bonds of enaminones. Inspired by the Reuben approach [87] energies have been related to two-bond deuterium isotope effects at carbons [96,97]. Recently, two-bond deuterium isotope effects (TBDIE) have been correlated to hydrogen bond energies in *o*-hydroxy aromatic aldehydes in which the hydrogen bond energies were calculated by the hb and out method [98]. The use of TBDIE has the advantage that no reference is needed. The hydrogen bond energies expressed as electron density at the bond critical point are plotted vs. two-bond deuterium isotope effects on ^13^C chemical shifts in Figure 25 for a small set of enaminones. The ring critical points were calculated using the B3LYP/6-311++G(d,p) functional [99] and the AIM program [100,101]. A reasonable correlation is obtained considering that both ketones, esters and nitro groups are acceptors and compounds are both linear and cyclic and substituents at nitrogen both aliphatic (methyl and *t*-butyl) and aromatic. It is obvious that the cyclic compounds fall on a line of their own.

Considering the correlation between the TBDIE on ^13^C and the electron density at the bond critical point (Figure 25) it is obvious that the large isotope effect is correlated to a stronger hydrogen bond. As TBDIE are also correlated to NH chemical shifts, a series of parameters may be used to predict hydrogen bond strength.

## 4. Hydrogen Bond Strength

**Figure 26:**
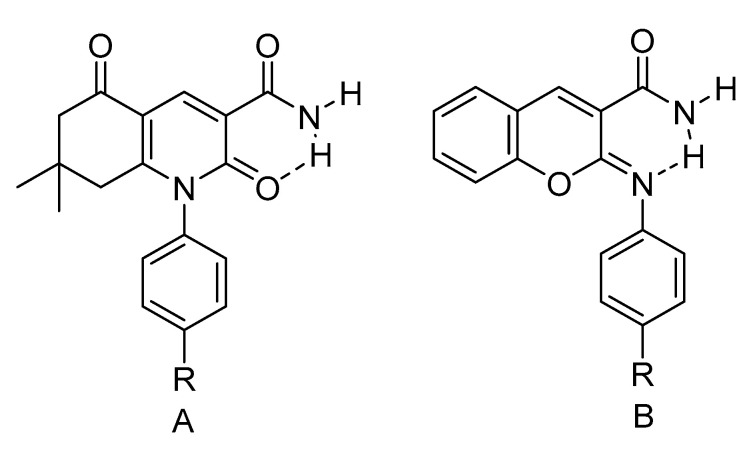
Primary amides used to estimate hydrogen bond strength. Taken from [105].

Hydrogen bond strength can be judged from the hydrogen bond energy (see Section 3). The work on predicting hydrogen bond strength using the pK_a_ slide should be mentioned as a reference method although not based on spectroscopic methods [104]. Gorobets et al. have suggested the difference between ^1^H chemical shifts of the chemical shifts of primary amides as a simple index of hydrogen bond strength in primary amides as demonstrated in Figure 26 [105]. The hydrogen bond strength can best be described by experimental trends like NH stretching frequencies, the lower the NH stretching frequency the stronger the hydrogen bond, for NH chemical shifts and two-bond deuterium isotope effects on ^13^C chemical shifts or one-bond deuterium isotope effects on ^15^N chemical shifts, the larger the stronger the hydrogen bond. A list of criteria also including structural parameters can be found in [106]. Martyniak et al. [107] also find that the asymmetry of the potential curve is a measure of hydrogen bond strength.

## 5. Assignments

### 5.1. CD Stretching Frequencies

A blue shift of C_δ_D_2_ stretching frequencies of prolines in the Src homology 3 domain was interpreted as a correlation with a hydrogen bond N_i+1_H…N_i_ interaction. The blue shifts were 10 and 17 cm^−1^ for Pro 165 and 21 and 28 cm^−1^ for Pro183 relative to two other prolines not affected. This was further supported by model studies of methyl terminated proline dipeptides similar to those of the SRC homology 3 domain. DFT calculations and NBO analysis supported this type of hydrogen bond [108].

### 5.2. Assignments

**Figure 27:**
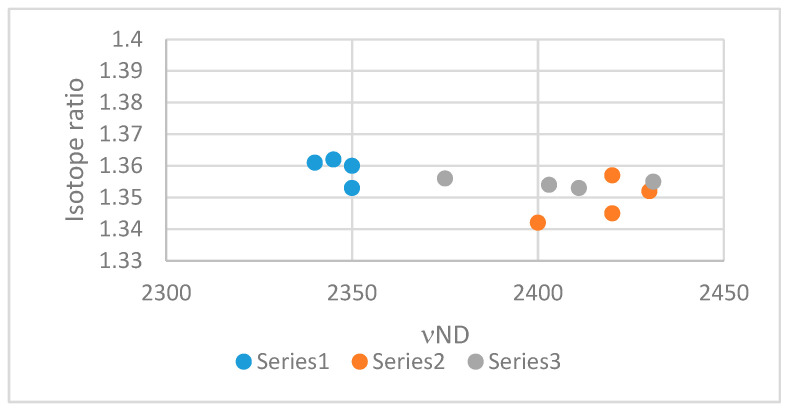
Plot of the νNH/νND ratio vs. νND. Series 1 β-amino-α-nitro-α,β-unsturated ketones, hydrogen bonding to the keto group, Series 2 Hydrogen bonding to the nitro group from [89] and Series 3 enaminoesters from [89,103].

As NH stretching vibrations can be difficult to assign, deuteriation is often a good tool. As most NH stretching frequencies are found around 3000 cm^−1^ the ND stretching frequencies will be around 2100 cm^−1^, which is in a region with few other resonances. However, the NH/ND ratio is not fixed. This was originally studied by Novak [109] and later supplemented by Sobczyk et al. [110] primarily using OH stretching frequencies. Novak included mostly intermolecular hydrogen bonds whereas Sobczyk et al. added intramolecular hydrogen bonds. The broad trend is a decrease of the isotope ratio as the NH stretching frequency decreases. However, as seen in Figure 27 based on data by Chiara et al. [89,103] for nitro-substituted enaminones and nitro-substituted enamino esters and supplemented with data on enamino esters the picture is not so clear cut at all. This is also seen in the review by Sobczyk et al. [73] but is to some extent masked by the inclusion of a correlation line.

## 6. Conclusions

In conclusion it is useful to deal with the three different types of intramolecular hydrogen bonds separately. For intramolecular hydrogen bonds of RAHB type hydrogen bond energies are difficult to obtain, resulting in very few or none experimental results are available. A useful method, although not tested to a large extent, on intramolecularly hydrogen NH…X systems, is electron densities at the ring critical point. The latter may also be correlated to deuterium isotope effects on ^13^C chemical shifts. In view of this, empirical parameters, NH chemical shifts, deuterium isotope effects on ^13^C or ^15^N chemical shifts may been used as indicators. NH chemical shifts have to be corrected for ring current effects, if the substituent at the nitrogen is an aromatic rings. Based on these parameters a large range of both donors and acceptors are investigated and rated with their ability to form intramolecular hydrogen bonds. NH chemical shifts and TBDIE have been correlated. However, NH chemical shifts have to be corrected for possible ring current effects, solvent effects and should be measured at as low concentration as possible. The TBDIE have the advantage of being measured as a difference and therefore being dependent on the just mentioned effects. Furthermore, deuterium isotope effects over hydrogen bonds may be used to identify hydrogen bonded pairs in case of multiple possibilities. The finding that ^1^*J*(N,H) is rather invariant makes this a good gauge for checking for tautomerism.

Charged systems with connecting bonds between donor and acceptor of the intramolecular hydrogen bonds, but no conjugation, show very large NH chemical shifts.

For intramolecular hydrogen bonds in proteins and nucleic acids coupling through the hydrogen bond can be measured and the magnitude increases with the shorter the heavy atom is. Also ^1^*J*(N,H) couplings are useful in the characterization of intramolecular hydrogen bonds.

Theoretical calculations are very useful in calculation of energies, coupling constants, isotope effects on chemical shifts and the finding that NH stretching frequencies can be calculated routinely means that they can be used more easily to identify infra red bands due to NH stretching vibrations. The use of νH/νD ratios to predict NH stretching frequencies based on ND stretching frequencies probably need more investigations.

## Data Availability

Not applicable.
